# Sex Distributions in the Most Frequent Autosomal Genetic Causes of Retinitis Pigmentosa

**DOI:** 10.1167/iovs.66.11.77

**Published:** 2025-08-29

**Authors:** Mark J. Hughes, Tina Lamey, Elena R. Schiff, Siying Lin, Terri Mclaren, Jennifer Thompson, Kirk A. J. Stephenson, Panagiotis Sergouniotis, Nikolas Pontikos, Malena Daich Varela, Mariya Moosajee, Ajoy Vincent, Michel Michaelides, Gavin Arno, Andrew R. Webster, Fred K. Chen, Omar A. Mahroo

**Affiliations:** 1 UCL Institute of Ophthalmology, University College London, London, England, United Kingdom; 2 Genetics Service, Moorfields Eye Hospital, London, England, United Kingdom; 3 Section of Ophthalmology, King's College London, St Thomas’ Hospital Campus, London, England, United Kingdom; 4 Australian Inherited Retinal Disease Registry and DNA Bank, Department of Medical Technology and Physics, Sir Charles Gairdner Hospital, Nedlands, Western Australia, Australia; 5 Division of Evolution, Infection and Genomics, The University of Manchester, Manchester, England, United Kingdom; 6 Manchester Centre for Genomic Medicine, Saint Mary's Hospital, Manchester University NHS Foundation Trust, Manchester, England, United Kingdom; 7 Centre for Ophthalmology and Visual Science (incorporating Lions Eye Institute), The University of Western Australia, Nedlands, Western Australia, Australia; 8 Department of Ophthalmology and Vision Sciences, The Hospital for Sick Children, University of Toronto, Toronto, Ontario, Canada; 9 Greenwood Genetic Center, Greenwood, South Carolina, United States; 10 Ophthalmology, Department of Surgery, University of Melbourne, Melbourne, Victoria, Australia; 11 Centre for Eye Research Australia, Royal Victorian Eye and Ear Hospital, Melbourne, Victoria, Australia; 12 Department of Physiology, Development and Neuroscience, University of Cambridge, Cambridge, United Kingdom

**Keywords:** retina, retinitis pigmentosa (RP), rod-cone dystrophy

## Abstract

**Purpose:**

The purpose of this study was to explore whether sex imbalances are detectable in the most frequent genetic causes of retinitis pigmentosa (RP).

**Methods:**

Databases from centers in three countries (Moorfields Eye Hospital, London; Hospital for Sick Children, Toronto; and Australian Inherited Retinal Disease Registry, Perth, Australia) were searched, quantifying numbers of male and female patients with disease attributed to variants in the six most frequently involved autosomal RP genes. Proportions of female patients (with 95% confidence intervals [CIs]) were calculated for each gene. Two-tailed binomial testing was performed (Bonferroni corrected threshold, *P* = 0.008) to investigate whether proportions differed significantly from an underlying male:female ratio of 1:1. For genes where the 95% CI did not include 50%, sex distributions were also explored in previously published cohorts.

**Results:**

Our search yielded 1454 patients with disease attributable to variants in *USH2A* (*n* = 550), *RP1* (*n* = 277), *RHO* (*n* = 246), *PRPF31* (*n* = 158), *EYS* (*n* = 124), and *MYO7A* (*n* = 99). Proportions of female patients (95% CI) for each gene were 46.2% (42.0–50.5%), 49.5% (43.4–55.5%), 55.3% (48.8–61.6%), 63.9% (55.9–71.3%), 39.5% (31.0–48.7%), and 42.4% (32.7–52.8%), respectively. The 95% CI did not include 50% for *PRPF31* and *EYS*; binomial testing revealed *P* values of 6.24 × 10^−4^ and 0.025, respectively. Combining with data extracted from previously published cohorts yielded *P* values of 1.62 × 10^−6^ and 0.0084, respectively.

**Conclusions:**

We observed a significant preponderance of female patients for *PRPF31*-associated RP and a preponderance of male patients in those with *EYS*-associated RP. Our findings suggest that sex is likely to be a modifier affecting penetrance in *PRPF31*-associated disease and might act in the opposite direction in disease associated with *EYS*.

A current area of increasing investigation is the search for modifiers in inherited retinal disease (IRD); patients who share the same pathogenic variants can experience disease of differing severity. Variability in penetrance (the proportion of individuals with the genetic variant(s) who are affected by the condition) and expressivity (the range or severity of clinical features displayed by those affected) are frequently observed, particularly in autosomal dominant disease. Identifying modifiers and understanding the mechanisms by which they act could potentially open new therapeutic avenues. Whereas sex imbalances in X-linked diseases are expected, sex has been shown to be a likely modifier in inherited eye conditions that are not X-linked. The male preponderance in mitochondrially inherited Leber Hereditary Optic Neuropathy has long been known, and has been shown even in autosomal causes of this disorder.^1^^,^^2^ Milder disease-associated variants in *ABCA4* appear to be more penetrant in female patients, whereas more severe variants appear to show similar penetrance in men and women.^3^^,^^4^ Our recent analysis of autosomal macular dystrophies showed a male preponderance in autosomal dominant Best disease, and a female preponderance in patients with *EFEMP1*-related dominant drusen.^5^

Retinitis pigmentosa (RP) is the most common IRD phenotype and can arise from variants in any one of a large number of genes. Incomplete penetrance and intrafamilial variability have been described for several RP genes, including *PRPF31* and others.^6^^–^^16^ In the present study, we explored sex distributions in autosomally inherited RP, as an imbalance might suggest sex is a modifier in some cases. To avoid very small numbers for each gene (i.e. to maximize power in our analyses), we focused on the most frequently involved genes. In our previous study of the large genetically characterized IRD cohort at Moorfields Eye Hospital,^17^^,^^18^ the most frequently encountered RP-associated autosomal genes were *USH2A*, *RP1*, *RHO*, *PRPF31*, *EYS*, and *MYO7A*. We therefore focused on these genes, and we also included cohorts based in other countries, namely the Australian Inherited Retinal Disease Registry^19^ and patients seen at the Hospital for Sick Children, Toronto, Canada. We quantified female patients and male patients with disease associated with each of these genes, aiming to establish whether a significant sex imbalance was evident.

## Methods

A retrospective search of the electronic patient record was initially done at Moorfields Eye Hospital in London. Patients referred for suspected IRD are examined by experienced retinal specialists, having detailed clinical history, ophthalmic examination, and imaging typically including spectral-domain optical coherence tomography (OCT) and short-wavelength fundus autofluorescence. Genetic testing was undertaken by various methods over the years, including sequencing of gene panels and, more recently, whole genome sequencing. This IRD cohort, the genetic testing strategies used and the demographic composition, have been described in more detail previously.^17^^,^^18^

In our prior study where we described the most frequently encountered genes in this cohort, we found that the top 20 genes accounted for more than 70% of families with a positive genetic diagnosis.^17^ In the present study, we chose from these 20 genes those associated with autosomal (dominant and recessive) RP; these were *USH2A*, *RP1*, *RHO*, *PRPF31*, *EYS*, and *MYO7A*. As the data from the previous study derived from a search conducted over 5 years ago, an updated search of the electronic patient record was performed in relation to these genes.^18^ *USH2A* is associated with both syndromic (type 2 Usher syndrome) and non-syndromic RP; and *MYO7A* is associated with type 1 Usher syndrome. For these genes, all patients affected with RP (both syndromic and non-syndromic) were included. *RP1* and *RHO* are typically associated with autosomal dominant inheritance. Any rare cases with bi-allelic variants (autosomal recessive) were excluded.

Additional data were collated from other cohorts. Ten patients with *PRPF31*-associated disease seen at the Manchester Centre for Genomic Medicine were added to the Moorfields cohort (the combined cohort is referred to in the Results section as the “UK cohort”). Numbers of male and female patients with disease associated with variants in the six genes seen at the Hospital for Sick Children, Toronto, Canada, were added (“Toronto cohort”) as well as numbers from the Australian Inherited Retinal Disease Registry (“Australia cohort”). Data were combined from all centers for each gene.

The proportion of female patients (with 95% confidence intervals [95% CIs]) was established for each genetic cause. This allowed identification of those genes for which the 95% CI did not include 50%, suggestive of a nominally significant imbalance. Furthermore, a two-tailed binomial test was performed to examine whether the proportion was significantly different from 50%: this allowed a *P* value to be generated. Whereas a *P* value < 0.05 is usually taken as nominally significant, we were conducting 6 separate tests; a Bonferroni correction yielded a corrected *P* value threshold of 0.0083.

Finally, for those genes for which 95% CI did not include 50% (i.e. those for which a nominally significant imbalance was found), a literature search was conducted for previously published cohorts from other centers. Studies were included with cohort sizes of 30 or more (for the relevant gene) and in which numbers of male patients and female patients were reported. Care was taken to avoid inclusion of multiple studies from the same center or region, to avoid counting individuals twice. Proportions of female patients (with 95% CI) were calculated for each cohort and binomial testing was again conducted combining data from the present study with published cohorts.

The study adhered to the tenets of the Declaration of Helsinki. Patients gave written informed consent for genetic testing. Research ethics approval was from the respective review boards (Moorfields Eye Hospital and the Northwest London Research Ethics Committee; REB 1000017804, Hospital for Sick Children; the human ethics committees at the University of Western Australia [2021/ET000151], and Sir Charles Gairdner Hospital [RGS04985], Perth, Western Australia, Australia).

## Results

### Combined Cohort

This study yielded 1454 patients with disease attributable to variants in the 6 genes of interest (including 868, 110, and 476 patients for the United Kingdom, Toronto, and Australia cohorts, respectively). Of the overall cohort, there were 719 female patients, equating to 49.4% (95% CI = 46.9–52.1%). Supplementary Table S1 gives a breakdown of male and female patients by cohort and by gene.

**Figure 1.**
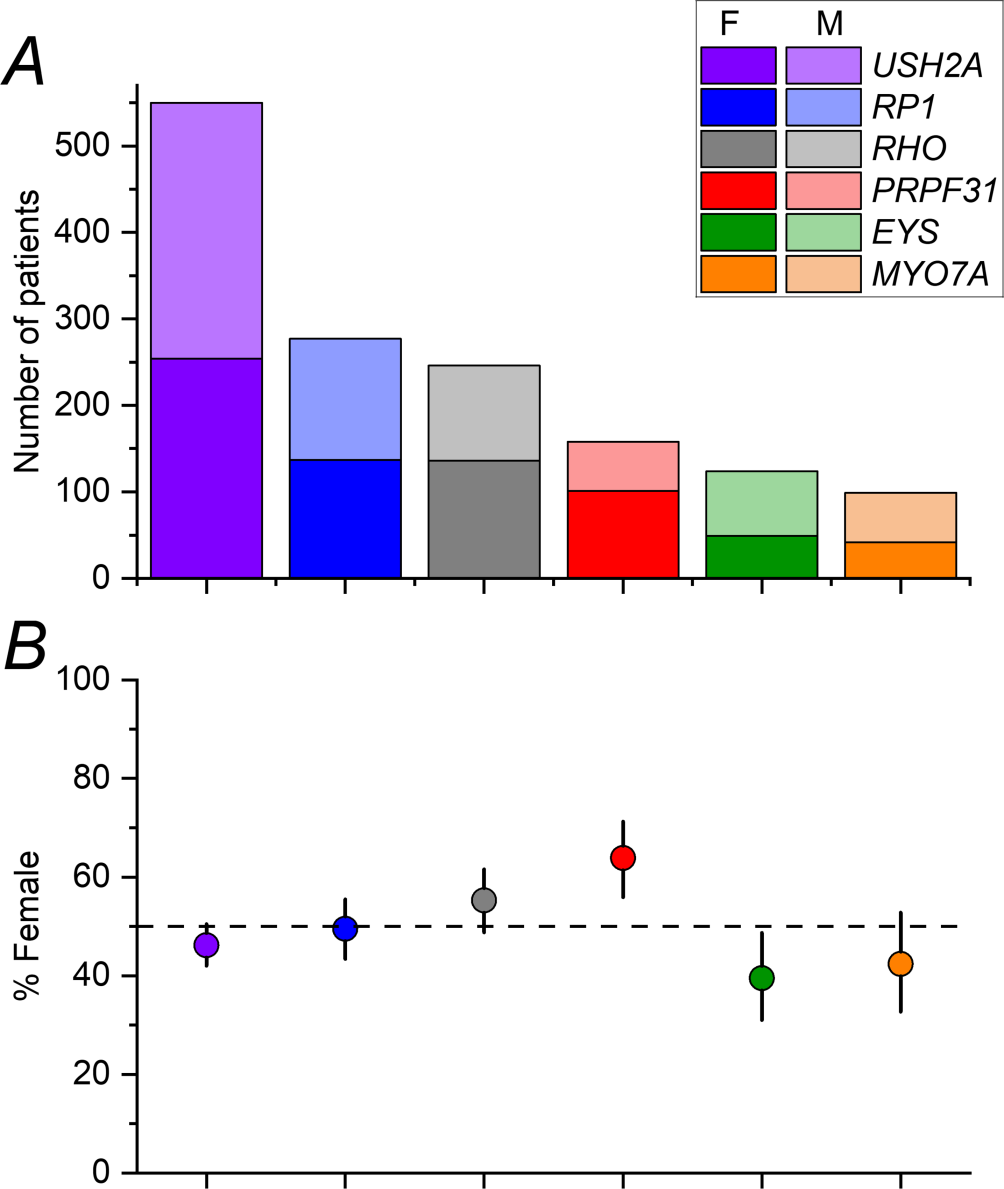
**Numbers of patients with each monogenic condition.** (**A**) Numbers of patients in each group (with darker and lighter portions of each bar representing female patients and male patients, indicated in the legend as F and M, respectively). (**B**) Proportion of female patients with 95% CI denoted by error bars. The *horizontal dashed line* shows 50%.

Numbers of patients with variants in *USH2A*, *RP1*, *RHO*, *PRPF31*, *EYS*, and *MYO7A* were 550, 277, 246, 158, 124, and 99, respectively. The Table lists the number of female patients and male patients for each condition, together with the significance level for the binomial test. Figure 1 shows overall numbers (upper panel) and proportions of female patients (lower panel). The error bars in the lower panel represent 95% CIs.

**Table.** Numbers and Sex Distributions for Autosomal RP Genes With Results of Two-Tailed Binomial Testing

		Numbers of Patients				
Gene	Mode of Inheritance	Total	Females	Males	Female % (95% CI)	*P* Value for Imbalance
*USH2A*	AR	550	254	296	46.2 (42.0–50.5)	0.0804
*RP1*	AD	277	137	140	49.5 (43.4–55.5)	0.904
*RHO*	AD	246	136	110	55.3 (48.8–61.6)	0.111
*PRPF31*	AD	158	101	57	63.9 (55.9–71.3)	6.24 × 10^−4^^*^
*EYS*	AR	124	49	75	39.5 (31.0–48.7)	0.0248
*MYO7A*	AR	99	42	57	42.4 (32.7–52.8)	0.159

AD, autosomal dominant; AR, autosomal recessive.

* *P* < 0.0083 denotes significance.

**Figure 2.**
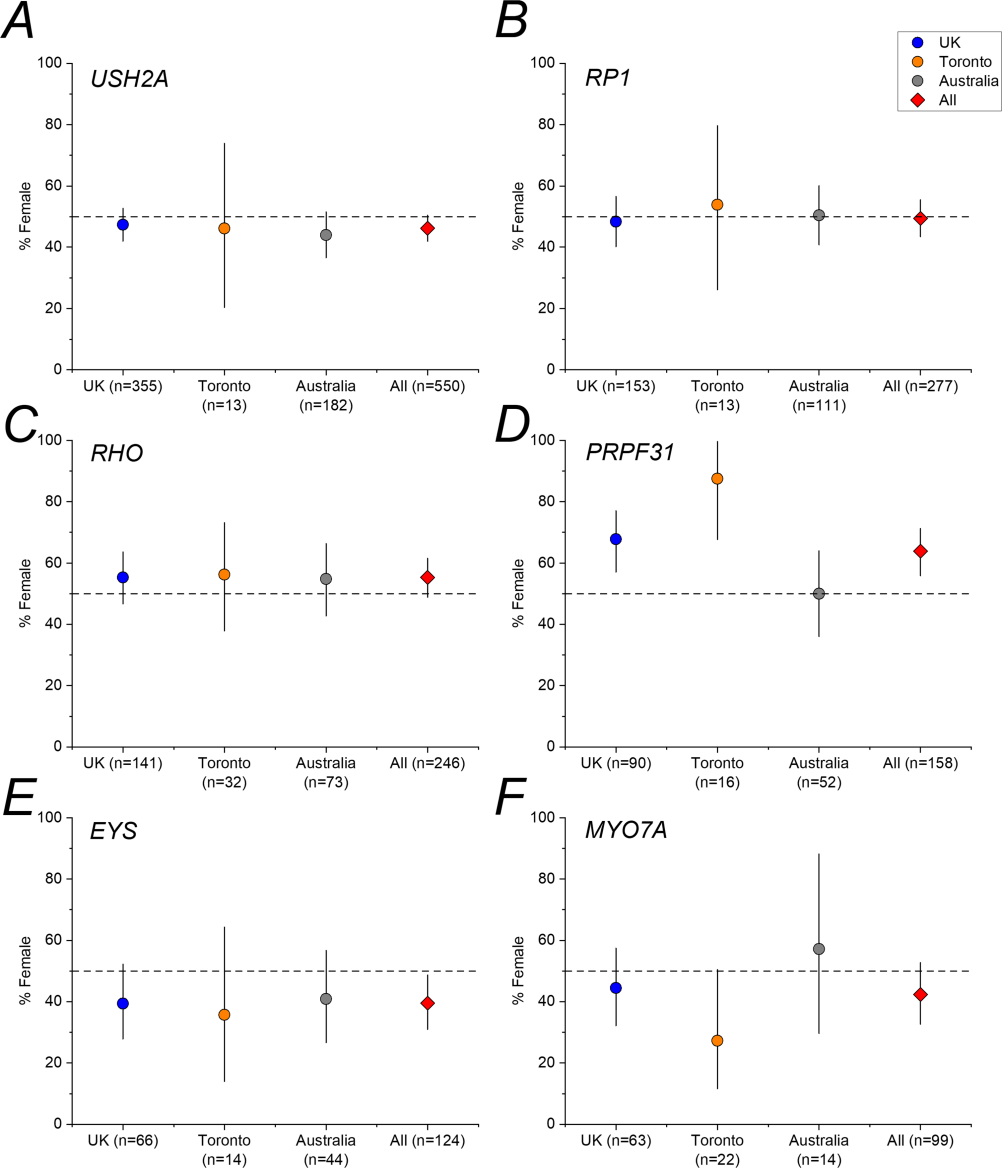
**Proportions of female patients for each gene within each of the three cohorts.** Error bars denote 95% CI. The *horizontal dashed line* shows 50%.

As evident in the Table and Figure 1, for 4 of the 6 genetic groups, the 95% CI included 50%. For *PRPF31*, a significant preponderance of female patients was observed. Binomial testing confirmed that the proportion of female patients for *PRPF31* was unlikely to be consistent with an underlying male-female ratio of 1:1 (2-tailed *P* value 6.24 × 10^−4^). For *EYS*, a preponderance of male patients was observed, with the upper limit of the 95% CI falling below 50%. Binomial testing yielded a 2-tailed *P* value of 0.0248, falling short of Bonferroni corrected significance. Figure 2 plots proportions of female patients for each gene within each cohort; for *EYS*, there appeared to be a consistent preponderance of male patients in each of the three cohorts.

### Selected Clinical Examples

**Figure 3.**
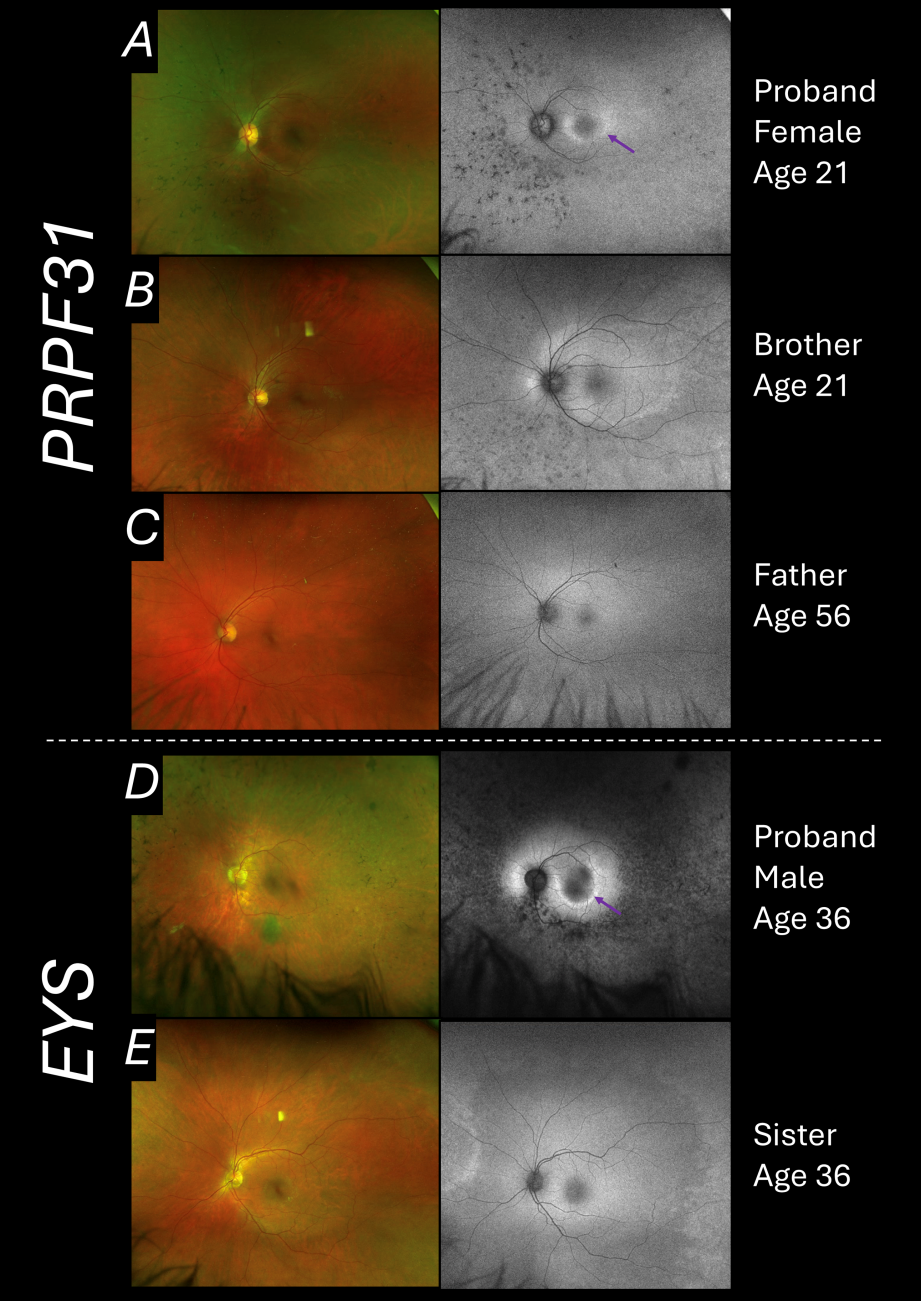
**Ultra-widefield fundus pseudocolor (*left panels*) and green wavelength autofluorescence (*right panels*) imaging from families with variants in *PRPF31* or *EYS*.** Images were acquired with the Optos system (Optos plc, Dunferminline, UK). Panels show images from the left eyes; findings from the right eyes were similar in all cases. (**A–C**) The three family members carry the same heterozygous frameshifting variant in *PRPF31*, p.(Thr258Glnfs*68). (**A**) Images from a 21-year-old female proband. The *purple arrow* highlights the hyperautofluorescent ring marking the outer boundary of preserved retina. (**B**) Images from the proband's brother showing milder degeneration at the same age. (**C**) Images from their father, showing no evidence of retinal degeneration. (**D, E**) Siblings carrying the same compound heterozygous pathogenic variants in *EYS*: p.(Ser761Ilefs*16) and p.(Cys385*). (**D**) Images from the male proband; the *purple arrow* highlights the partial hyperautofluorescent ring marking the outer boundary of preserved retina. (**E**) Images from the proband's sister taken at the same age, showing milder, more peripheral retinal changes, without the macular hyperautofluorescent ring.

Figure 3 shows fundus imaging from two families to illustrate examples of variability in the phenotype for the two genes that showed apparent imbalances. The left panels show pseudocolor images, reflecting the clinical appearance; the right panels show green wavelength autofluorescence images, which can better highlight the extent of retinal degeneration. Figure 3 panels A to C are from a family with a frameshifting variant in *PRPF31*. The proband (Fig. 3A) was a 21-year-old woman with visual symptoms (night blindness and peripheral field loss). Imaging shows peripheral atrophy and the hyperautofluorescent ring (characteristically seen in RP) which delineates the boundary between intact retina (within the ring) and degenerated outer retina (outside the ring). Interestingly, her brother, who carried the same variant, did not have visual symptoms and imaging (acquired at the same age as for his sister) shows a markedly milder, more peripheral degeneration (Fig. 3B). Their father, who shared the variant, was asymptomatic and had no signs on examination or imaging (Fig. 3C).

Figures 3D and 3E depict images from a brother and sister with the same compound heterozygous variants in *EYS*. Images acquired from the brother (Fig. 3D) show a marked degeneration. A hyperautofluorescent ring is evident (deficient superiorly, similar to some previous reports in *EYS*-associated disease).^20^^,^^21^ His symptoms began 11 years prior to these images. Figure 3E shows images from his sister taken at the same age; she was asymptomatic (only developing some photopsia some years later) and the abnormalities on imaging are milder and much more peripheral.

### Numbers in Previously Published *PPRF31* and *EYS* Cohorts

**Figure 4.**
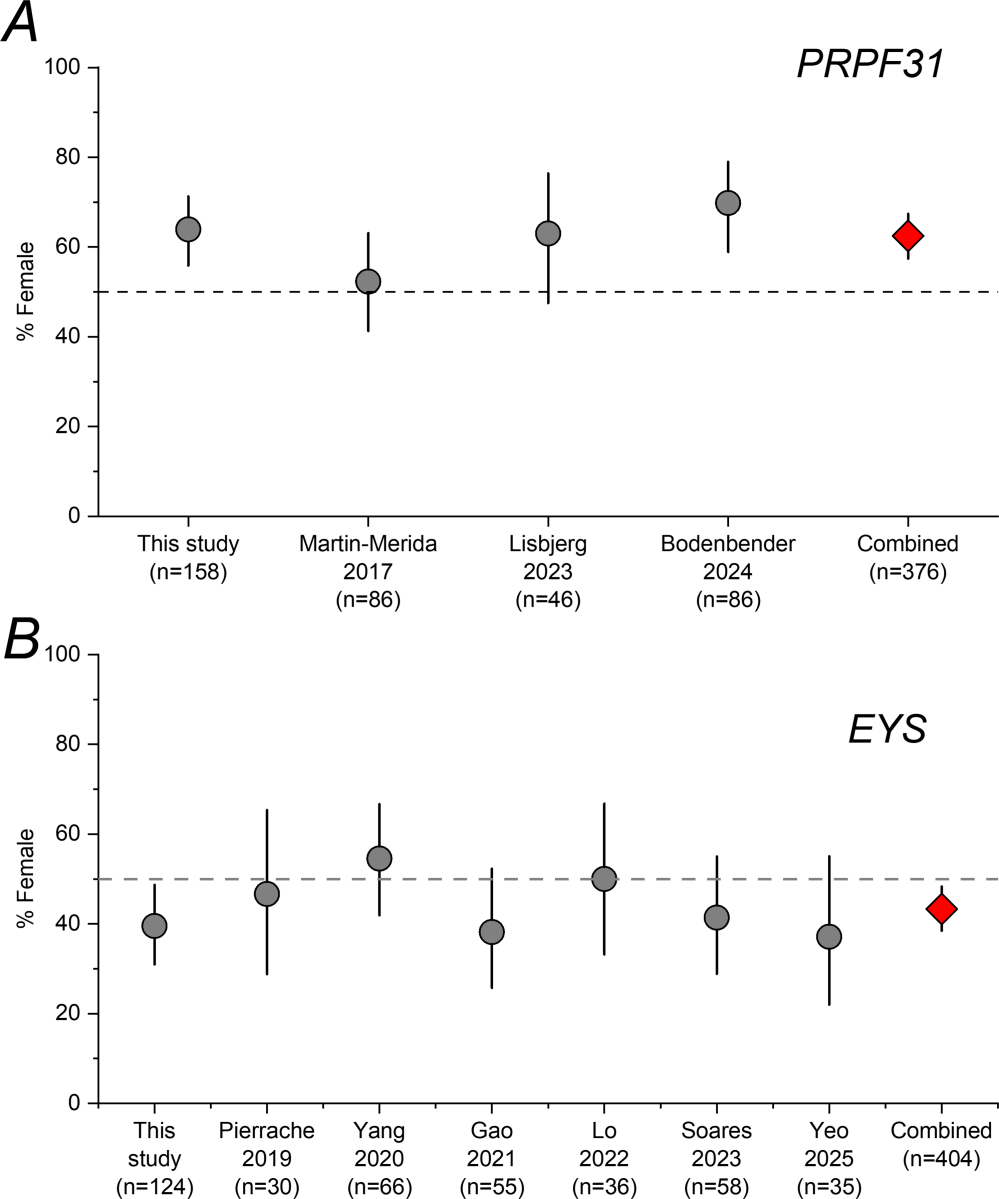
**Proportions of female patients in cohorts of patients with *PRPF31*-associated and *EYS*-associated RP reported in the literature.** Error bars show 95% confidence intervals. The *dashed line* indicates 50%. *Red diamond symbols* combine data from the previously published cohorts with the present study. (**A**) Cohorts of patients with *PRPF31*-associated RP. (**B**) Cohorts of patients with *EYS*-associated RP.

Given our findings, we sought to examine sex distributions in previous reports of patients with *PRPF31*-associated RP. Most reports in the literature constitute relatively small numbers of patients, often single pedigrees. Some studies of larger cohorts did not report numbers of male and female patients. Prior studies did not specifically explore sex imbalances. We found three publications in the literature, each reporting on more than 30 patients, with sex composition included. A study from Spain, published in 2017,^22^ presented data from 15 pedigrees, with affected individuals highlighted: of 86 reportedly affected individuals for whom sex was known, 45 were female patients. A later study of a Nordic cohort^23^ presented data from 46 patients, of whom 29 were female patients. A recent publication from a German cohort included 86 patients,^24^ of whom 60 were female patients. In that study, female sex was found to be associated with cystoid macular edema. Figure 4A plots proportions of female patients (with 95% CIs) extracted from these studies, together with the proportion in the present study, and the overall proportion when combining all 4 cohorts (62.5%, 95% CI = 57.4–67.4%). A consistent preponderance of female patients is apparent, with binomial testing for the combined group reaching strong statistical significance (2-tailed *P* value, 1.62 × 10^−6^).

We also explored previously published cohorts of patients with variants in *EYS*. We found six prior studies, reporting cohorts from the Netherlands (*n* = 30 patients; 14 female patients),^25^ Japan (*n* = 66; 36 female patients),^26^ China (*n* = 55; 21 female patients),^27^ Taiwan (*n* = 36; 18 female patients),^28^ Portugal (*n* = 58; 24 female patients),^29^ and Singapore (*n* = 35; 13 female patients).^30^ Four of these cohorts had a majority of male patients (for the remaining 2 cohorts, there were equal numbers or more female patients). Proportions of female patients are plotted in the lower panel of Figure 4. Combining with the present study yielded an overall proportion of female patients of 43.3% (95% CI = 38.5%–48.3%). Binomial testing yielded a 2-tailed *P* value of 0.00837. A recently published study that included patients from Singapore and Japan also had a preponderance of male patients (*n* = 74 patients; 34 female patients).^21^ However, we excluded this due to likely overlap with the previously published cohorts from Singapore and Japan which were already included. (Inclusion of this study would have yielded an overall *P* value of 0.00696.)

## Discussion

In this exploratory study, we investigated sex distributions in patients with the most frequently encountered autosomal genetic causes of RP (grouped by gene). For four of the six genes, no significant imbalance was observed (the 95% CI for the proportion of female patients included 50%). For *PRPF31*-associated RP, we found a preponderance of female patients (comprising 64% of these patients); binomial testing indicated that our results were significantly different from that expected with an underlying male:female ratio of 1:1 (2-tailed *P* value, 6.24 × 10^−4^). For *EYS*, we observed an imbalance in the opposite direction (preponderance of male patients), which was nominally significant (*P* = 0.025), but did not meet the significance threshold after correction for multiple testing. Combining with data extracted from previously published geographically diverse cohorts appeared to increase the level of significance for both imbalances (*P* = 1.62 × 10^−6^ and 0.0084 for *PRPF31* and *EYS*, respectively).

Variants in *PRPF31* are a frequent cause of autosomal dominant RP, and incomplete penetrance is a well-reported feature, the underlying mechanisms of which are still not fully understood.^6^^–^^12^ Incomplete penetrance is not typically observed to such an extent for the other genes. Expression levels of the non-mutant *PRPF31* allele are thought to contribute, but it is likely that other modifiers also exist. The present study suggests that sex is a potential modifier in *PRPF31*-associated disease. The family depicted in Figure 3 shows an example of the variability that can be observed: here, the female proband is more severely affected, whereas the male family members carrying the same variant show milder or no disease.

Potential mechanisms for sex imbalances in *PRPF31*-associated disease are unclear. It remains unknown whether sex influences disease penetrance, or disease severity leading to the increased presentation and diagnosis of female carriers of *PRPF31* pathogenic variants. If female sex is a risk factor for cystoid macular edema,^24^ then it is possible that women may be more likely to seek ophthalmic review owing to impairment in central vision (which might be more noticeable or debilitating than the impaired night vision that usually constitutes the first symptom in RP), even if male and female patients had the same underlying risk of developing RP. However, in the example shown in Figure 3 (one family and hence not necessarily reflective of the full cohort), the extent of peripheral degeneration clearly differs between the siblings (with images captured at the same age), indicating more than a difference in degree of macular involvement.

For *EYS*, non-penetrance is not so typical, but variability is observed in extent and in patterns of degeneration between patients. Our study raises the intriguing possibility that sex might act as a modifier (in the opposite direction from that seen in *PRPF31*), with men more severely affected. One study has suggested, based on retinal organoid data, increased susceptibility to phototoxicity as a possible contributory mechanism of degeneration in *EYS*-associated disease.^31^ One might hypothesize that, in some populations at least, men have greater light exposure (or that male retinas are more susceptible even with the same degree of light exposure). Data reflecting real-life light exposure in the general population (obtained from wrist-worn devices in a large US-based population study) have indeed suggested that women have substantially less bright light exposure compared with men.^32^ This might also differ between populations, and so if this does have relevance to *EYS-*associated degeneration, such differences might contribute to variability in sex imbalances seen in different cohorts (Fig. 4B).

Sex differences in retinal disease incidence and severity could arise from both biological and behavioral factors, and their interactions. Structurally, retinal thickness differs between the sexes in healthy individuals.^33^^–^^35^ It is also possible that sex hormones affect retinal physiology, acting as a potential modifier in disease^36^:^36^ messenger RNAs for estrogen, progesterone, and androgen receptors have been identified in retinal cell types.^37^^,^^38^

Potential limitations of our study bear mention. As IRDs are individually rare, sample sizes are inevitably small, limiting overall power, particularly for the rarer conditions. Our main study cohort derived from three centers, and so might not be generalizable to other populations, although we found broadly similar findings when analyzing several published *PRPF31* and *EYS* cohorts from other countries. In addition, the precise ethnic makeup of the cohorts was not available, although previous data from the Moorfields Eye Hospital cohort have shown it to be multi-ethnic.^17^ Even in ethnically diverse cohorts, environmental factors might be similar for a given geographic location and so findings might not be applicable to other settings.

Another important limitation is that genotype within each gene was not explored. It is possible that sex may act as a modifier for certain genetic variants and not for others (the previous study of *ABCA4*-retinopathy highlighted sex imbalances for milder variants and not for patients with severe bi-allelic variants).^3^^,^^4^ This might also explain why sex imbalances were not as prominent in some cohorts (as seen in Fig. 4) as the prevalence of specific variants differs between populations. For the present study, patients were not sub-grouped by variant given the likely resulting loss of power and need to further correct for multiple testing. Future multi-center studies, for example, each focusing on a single gene, could investigate this.

Future studies could also look at important additional parameters that would be relevant to disease penetrance and severity, including age at onset of symptoms, age at diagnosis, rate of progression of degeneration, area of affected retina or visual field, presence of macular oedema, and several other variables that could be extracted from the medical history or from retinal imaging and functional studies (the latter could include psychophysical function and electroretinography). These data were not readily extractable for the current cohorts in sufficiently large numbers. Future investigations could incorporate statistical analyses that include some of these factors as covariates to explore in greater detail the extent to which sex might influence disease phenotype and progression. Another approach could be to systematically examine variability within families^39^;^39^ again, achieving sufficiently large numbers is likely to be a challenge.

Selection bias is possible. Sex differences can emerge from underlying sex differences in population structure and also differences in propensity to seek healthcare.^40^^,^^41^ In some contexts, healthcare may not be universally accessible, and should this differ by sex, this would also affect the results (where a lower proportion is observed for the sex less likely, or able, to access medical services). For the UK and Toronto cohorts, genetic testing is nationally funded and so is in principle freely accessible to all patients. However, in the past, and in many settings currently worldwide, funding for genetic testing (as well as the testing strategy, including the range of genes covered) has varied: any difference by sex in likelihood of achieving a molecular diagnosis might also contribute to ascertainment bias and affect observed proportions. Even where genetic testing does not pose direct financial costs to patient, there may be many other practical and logistic factors that affect referral and access to specialist review (and therefore testing) that might differ by sex. Such factors, however, might be expected to affect all genetic RP subtypes similarly, whereas we found imbalance specifically for only two of the genes, and indeed in opposite directions.

Sex imbalances are well-reported in many acquired retinal diseases, including macular holes and other vitreomacular interface disorders (more common in women),^42^^,^^43^ retinal tears and detachments (more common in men),^44^^,^^45^ and age-related macular degeneration (female preponderance).^46^ Such imbalances might arise from several mechanisms (given that many of the diseases, including IRDs, act via diverse pathways). Sex imbalances in autosomal Mendelian non-retinal diseases are also being explored.^47^ The present study identifies sex as a potential modifier in *PRPF31*-associated RP and possibly also for *EYS*-associated RP. Our study invites replication and future investigations of larger, multicenter patient cohorts so that rarer genetic causes of RP might be similarly investigated with sufficient power.

## Supplementary Material

Supplement 1
