# Supplementary material for: Sex Distributions in the Most Frequent Autosomal Genetic Causes of Retinitis Pigmentosa
Source: Invest Ophthalmol Vis Sci. 2025 Aug 29;66(11):77. doi: 10.1167/iovs.66.11.77 (PMC12400967; doi:10.1167/iovs.66.11.77)
Supplement: Supplement 1 [file iovs-66-11-77_s001.pdf]

Gene	Category	UK	Toronto	Australia	Combined
USH2A	*Total*	*355*	*13*	*182*	*550*
	Females	168	6	80	254
	Males	187	7	102	296
RP1	*Total*	*153*	*13*	*111*	*277*
	Females	74	7	56	137
	Males	79	6	55	140
RHO	*Total*	*141*	*32*	*73*	*246*
	Females	78	18	40	136
	Males	63	14	33	110
PRPF31	*Total*	*90*	*16*	*52*	*158*
	Females	61	14	26	101
	Males	29	2	26	57
EYS	*Total*	*66*	*14*	*44*	*124*
	Females	26	5	18	49
	Males	40	9	26	75
MYO7A	*Total*	*63*	*22*	*14*	*99*
	Females	28	6	8	42
	Males	35	16	6	57

Supplementary Table 1. Numbers of male and female patients for the six genes in the three cohorts. The "UK cohort" mainly comprised patients seen at Moorfields Eye Hospital in London, but also included 10 patients (8 females, 2 males) with *PRPF31*-associated disease from the Manchester Centre for Genomic Medicine.
